# Preliminary Evaluation of New Wearable Sensors to Study Incongruous Postures Held by Employees in Viticulture

**DOI:** 10.3390/s24175703

**Published:** 2024-09-02

**Authors:** Sirio Rossano Secondo Cividino, Mauro Zaninelli, Veronica Redaelli, Paolo Belluco, Fabiano Rinaldi, Lena Avramovic, Alessio Cappelli

**Affiliations:** 1 Department of Human Science and Quality of Life Promotion, Università Telematica San Raffaele Roma, Via di Val Cannuta 247, 00166 Rome, Italy; sirio.cividino@uniroma5.it (S.R.S.C.); veronica.redaelli@uniroma5.it (V.R.); alessio.cappelli@uniroma5.it (A.C.); 2 Lake Research and Study Centre, Via Vittor Pisani 8, 20100 Milan, Italy; fabiano.rinaldi@crslaghi.net (F.R.); lena.avramovic@crslaghi.ne (L.A.); 3 LTW3, Via Caduti di Marcinelle 7, 20134 Milano, Italy; paolo.belluco@lwt3.com

**Keywords:** viticulture, risk assessment, ergonomic, safety in agriculture, wearable sensors

## Abstract

Musculoskeletal Disorders (MSDs) stand as a prominent cause of injuries in modern agriculture. Scientific research has highlighted a causal link between MSDs and awkward working postures. Several methods for the evaluation of working postures, and related risks, have been developed such as the Rapid Upper Limb Assessment (RULA). Nevertheless, these methods are generally applied with manual measurements on pictures or videos. As a consequence, their applicability could be scarce, and their effectiveness could be limited. The use of wearable sensors to collect kinetic data could facilitate the use of these methods for risk assessment. Nevertheless, the existing system may not be usable in the agricultural and vine sectors because of its cost, robustness and versatility to the various anthropometric characteristics of workers. The aim of this study was to develop a technology capable of collecting accurate data about uncomfortable postures and repetitive movements typical of vine workers. Specific objectives of the project were the development of a low-cost, robust, and wearable device, which could measure data about wrist angles and workers’ hand positions during possible viticultural operations. Furthermore, the project was meant to test its use to evaluate incongruous postures and repetitive movements of workers’ hand positions during pruning operations in vineyard. The developed sensor had 3-axis accelerometers and a gyroscope, and it could monitor the positions of the hand–wrist–forearm musculoskeletal system when moving. When such a sensor was applied to the study of a real case, such as the pruning of a vines, it permitted the evaluation of a simulated sequence of pruning and the quantification of the levels of risk induced by this type of agricultural activity.

## 1. Introduction

Musculoskeletal disorders (MSDs) can arise in all types of activities, tasks, or sectors, such as agriculture, [1,2,3] and industry [3,4,5]. Every year, many farmers suffer from work-related diseases or accidents [6,7]. Actions to increase their safety are therefore necessary.

Viticulture presents significant risks of MSDs because many tasks are manual. Harvesting, pruning, shoot removal, and tying are only some the examples of manual activities frequently involved in vine practice [6,8]. Pruning with pruners or knives requires carrying out repetitive and powerful movements that may cause biomechanical stresses on the upper extremities and increase the risk of MSDs [9]. The opening span of common pruners may not match with the hand anthropometry of workers. As a result, workers must apply additional force to prevent wide opening of the handles [10,11] and may consequently be exposed to a bigger risk of musculoskeletal injury due to the further load required [11]. More generally, MSDs in vineyards are often caused by iterative forces applied to upper extremities or trunk, or back bends, twisting, and repetitive lifting of excessive loads [10,11].

Many ergonomic technologies have been developed and tested to limit MSDs. These technologies generally aim to optimize the interfaces between worker and workplace, to improve tools, as well as to investigate how to reduce excessive force, repetitive motions, and uncomfortable postures. In fact, it is common knowledge that small improvements to work tools or movement sequences can significantly reduce reported pain [12]. As a successful example of this, we can report the use of tubs of a smaller size in the harvesting of grapes [13].

In order to study uncomfortable working postures and to prevent MSDs, many risk assessment methodologies have been explored, including: OCRA (Occupational Repetitive Action) [14], REBA (Rapid Entire Body Assessment) [15], RULA (Rapid Upper Limb Assessment) [10], NIOSH (National Institute for Occupational Safety and Health) [16], and MAPO (Movement and Assistance of Hospital Patients) [17]. In agriculture, the RULA, OCRA, and REBA methodologies [18] are the most frequently used. Among these, the RULA method stands out for its ability to swiftly analyze hand–arm movements and assess ergonomic risks [19,20].

The RULA method was developed to provide a rapid evaluation of individual workers’ exposure to ergonomic risk factors associated with upper extremity MSDs. This ergonomic assessment tool considers the biomechanical and postural demands placed on the neck, trunk, and upper limbs by job tasks. RULA employs a systematic process to assess the required body posture, the force exertion, and the repetition associated with the task being evaluated [21]. A single-page worksheet is used to investigate body posture, muscle use frequency, and power exertions. The OCRA method was proposed for analyzing workers’ exposure to upper limb risk factors (repetitiveness, force, uncomfortable postures and movements, lack of recovery periods, and others, defined as “additional”). The REBA method [15,22] is a systematic process used to assess the risks of MSDs for the whole body, and the risks due to the ergonomic design related to the work. The result of REBA is a single score that shows the level of MSD risk for the evaluated job task. The minimum REBA score is 1, while the maximum is 15 [15,18,22].

**Figure 1:**
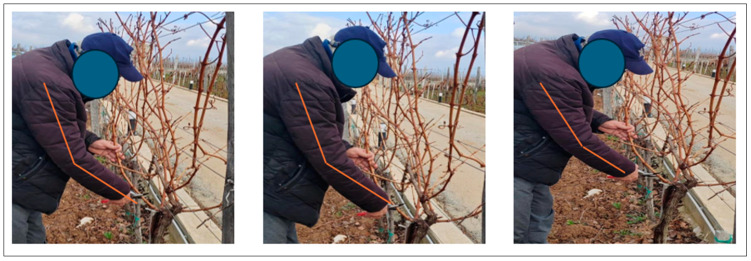
Semi-direct methods: example of application of the data acquisition method through a sequence of images [3].

Existing methodologies are generally based on checklists and analyses of short videos recording workers’ activities (or images, Figure 1). They are called semi-direct methods [3] and they are usually oriented to different MSD risk factors [3,23]. They cannot continuously monitor workers’ postures, and they do not consider job requirements and workers anthropometric characteristics [24]. They do not require the use of sensors or measuring instruments to acquire workers’ movements. As a result, they are generally applied to few cases, limiting their effectiveness.

Numerous sensors have been developed to acquire workers’ movements and physiological data and to propose analytical methods for occupational safety, primarily linked to the industrial sector [25,26]. Overall, they are wearable sensors for ergonomic assessment, ranging from sensor-equipped shirts [27] that can detect ergonomic and occupational safety aspects [28] to integrated production systems [29,30,31,32,33,34] like de DorsaVi ViSafe^+^. In order to provide some examples, we will list the following classification:

- *Inertial Measurement Units (IMUs).*
  These sensors, consisting of accelerometers, gyroscopes, and magnetometers, are utilized to perform quantitative biomechanical risk assessments. They are often employed to analyze work activities and to identify risks associated with upper limb movements and postures. The ZurichMOVE 1 is an example of a commercial IMU, widely used in gait monitoring. This sensor facilitates the collection of data such as axial accelerations and angular velocities, which are key biomechanical assessment parameters for activities such as walking or running.
- *Surface Electromyography (sEMG).*
  Employed to monitor muscle activity, sEMG sensors are often used to assess muscular fatigue and biomechanical loads during work tasks. These sensors provide direct data about muscle activity, facilitating the classification of work activities into low-risk and high-risk categories. Delsys Trigno is a well-known commercial sEMG that is widely used in the research for assessing muscle activity. It is acknowledged for being wireless and for providing a high signal quality, thus being suitable for both laboratory and field studies.
- *Pressure and Feedback Sensors.*
  Integrated with human augmentation technologies, these sensors enhance workers’ awareness of potential risks and improve safety and effectiveness within manual handling tasks. The Novel Pedar system is a pressure measurement system typically used in footwear to assess pressure distribution and loading patterns during activities. It is useful both in clinical settings and in research to evaluate biomechanical loads on the lower limbs.

The cited wearable sensors have also been used for research purposes, achieving remarkable results; however, their use could be difficult to replicate in the agricultural [35] and vine sectors.

Here is how a wearable sensor for ergonomic analysis should be in the agricultural [35] and viticultural sectors:

- *Cheap.*
  Agricultural tasks are often difficult to standardize as they are influenced by variables such as weather conditions and plant layout on the vineyard, or characteristics of the training system. In order to acquire useful data, it is mandatory to monitor a large group of workers who, in the case of a pruning team, could number up to 10 or 12 workers [35]. For all of these reasons, wearable sensors for ergonomic analysis that focus on agricultural and viticultural sectors should be cost-effective, in order to allow the real-time monitoring of several operators.
- *Robust.*
  Agricultural instruments must withstand external conditions, such as rain and mud, as well as accidental impacts. For these reasons, wearable sensors for ergonomic analysis that focus on the agricultural and viticultural sectors should be robust in order to ensure their durability under adverse conditions and their functionality in case of accidental collisions.
- *Adaptable and Easy to use.*
  Farmers have different anthropometric characteristics and are generally involved in seasonal activities that are required to be completed in a short time. Therefore, wearable sensors, used to perform ergonomic analysis in a large group of farmers in a real scenario, should be easily adaptable to many anthropometric characteristics, as well as easy to use in order to keep up with agricultural activities. Furthermore, they should not need extensive further data processing, so as to give rapid feedback about injury risk assessments. Even if some emerging technologies, such as AI and machine learning, could facilitate data processing to assess biomechanical load, also within agriculture and during harvesting phases, their use does not seem to be easily applicable to the viticultural sector where the operator’s hand could be obscured by vegetation, making such technology less useful [36].

The abovementioned wearable sensors are generally expensive commercial tools, most of the time devised for some indoor or laboratory applications that may require a long and difficult set-up. As a consequence, here is what is missing in the wearable sensor field: low-cost movement acquisition systems that can work outdoors and resist accidental collisions, and a versatile design to fit all anthropometric characteristics of workers, in order to study biomechanical risks and to prevent future MSDs in the agricultural and viticultural sectors.

The aim of this research was the development of a new technology to collect accurate data about uncomfortable postures and repetitive movements typical of viticulture workers. In order to reach this goal, two specific targets were established. The first target was the development and testing of a new wearable device capable of measuring real-time data about wrist angles and workers’ hand positions, during possible viticultural operations [22,28,37,38,39,40]. The main underlying requirements of the project were the development of a low-cost measurement system robust enough to be used in viticultural activities, easily adaptable to the different anthropometric characteristics of workers, and useful to prevent possible MSDs. Then, the second target was testing the resulting measurement system so as to evaluate incongruous postures and repetitive movements of workers’ hand positions during simulated pruning operations in vineyards [11,13,40], according to conventional methods such as REBA, RULA, and OCRA.

## 2. Materials and Methods

The study was divided into two phases:

1. the development and testing of a new wearable sensor (or measurement system);
2. the application of the measurement system developed in a real case (i.e., the pruning of a vine).

During the first phase of the study, after having developed the sensor, its reliability was checked. Many measurements at different reference angles were acquired by the sensor. The precision of the collected data was evaluated through a dedicated statistical analysis.

In the following phase of the research, the developed sensor was applied to a real case (i.e., the pruning of a vine). The pruning of a vine was simulated to evaluate the ergonomics of the gesture. On the basis of data collected through the measurement system, the risk factors were evaluated. To evaluate risk factors, different risk thresholds were classified (i.e., different limit angles for the hand–wrist–arm system) on the basis of regulations, scientific literature, and ergonomic risk assessment methods [10,16,41].

### 2.1. Phase 1: Development and Testing of the Measuring System

#### 2.1.1. Development of the Wearable Sensor

Here are the main requirements met during the development of the sensor: (a) a good relationship between costs and performance; (b) good robustness; (c) great “wearability” in order to easily record kinetic data from different parts of the body, in particular from the hand–wrist–arm system.

**Figure 2:**
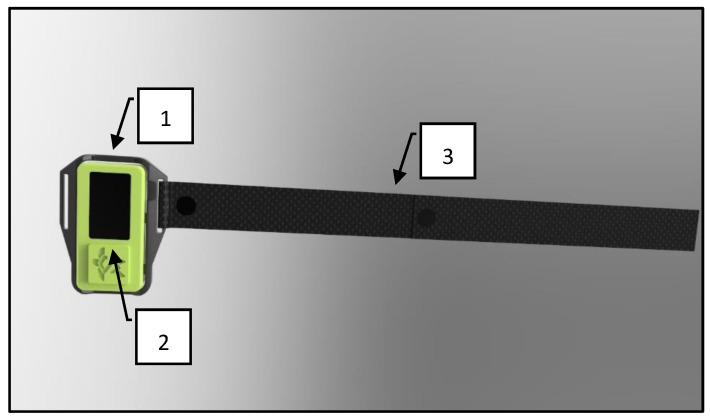
Example parts of the developed sensor: (**1**) the design of the 3D printed sensor, aiming at the maximum contact surface with the body; (**2**) the display, used to show the real-time data acquired during the test; (**3**) the whip straps, added to the case to facilitate its wearing.

To meet the cited requirements, the sensor was based on a low-cost ESP32 developing board. It had 3-axis accelerometers and gyroscope. This hardware allowed us to record the sensor positions in the space and its kinetics during its movements. The sensor housing was built with a 3D printer. Its design was devised in order to obtain good robustness and the maximum contact surface with the body. A whip strap was added to the case to facilitate the wearing of the sensor (Figure 2).

The sensor firmware was set up by an Arduino platform. The acquired data were sent via Bluetooth to an external device (e.g., a smartphone to which the sensor had previously been paired). However, the real-time acquired data were always available, because the three axes (X, Y, and Z) were graphically reported on a small screen LCD, mounted on the top of the sensor.

#### 2.1.2. Processing of Raw Data Collected by a Sensor

In order to calculate the position of a sensor in the space, raw data collected by accelerometers and gyroscopes were considered in the post-processing phase. All steps performed and mathematical formulas employed in the data processing were as follows.

**Preparation of the accelerometer data.**

The sensor measured the acceleration along three cardinal axes (x, y, z). To calculate its real acceleration (*a_real_*), excluding gravity, the following formula was used:

(1) $$a_{real}=a_{measurament}\;-\;g$$

where:

- $a_{measurament}$ is the acceleration measured by the sensor,
- $a_{real}$ is the acceleration of the device excluding gravity,
- g is the acceleration of gravity.

**Integration of acceleration to calculate the velocity of the sensor.**

To calculate the velocity of the sensor (*v*) through its acceleration (*a_real_*), the following formula was used:

(2) $$v\left(t\right)=v\left(t-1\right)+\int_{t-1}^{t}a_{real}\left(t\right)d\left(t\right)$$

where:

- v(t) is the velocity of the sensor at time t,
- v(t−1) is the velocity of the sensor at the previous time,
- $a_{real}(t)$ is the acceleration corrected for orientation and free from the gravity effect.

**Integration of velocity to calculate position of the sensor.**

To calculate the position of the sensor (*p*) through its velocity (*v*), the following formula was used:

(3) $$p\left(t\right)=p\left(t-1\right)+\int_{t-1}^{t}v\left(t\right)d\left(t\right)$$

where:

- $p\left(t\right)$ is the position of the sensor at time t,
- $p\left(t-1\right)$ is the position of the sensor at the previous time,
- $v\left(t\right)$ is the sensor at time t.

**Calculation of the orientation of the sensor through gyroscope data.**

The gyroscopes measured the angular velocity (*ω*) around the three cardinal axes (x, y, z). The orientation of the sensor was calculated integrating the angular velocity, recorded over the time, by the following formula:

(4) $$\theta \left(t\right)=\theta \left(t-1\right)+\int_{t-1}^{t}\omega \left(t\right)d\left(t\right)$$

where:

- $\theta \left(t\right)$ is the orientation of the sensor at time t,
- $\theta \left(t-1\right)$ is the orientation of the sensor at the previous time,
- ω is the angular velocity measured by gyroscopes,
- Δt is the time interval between measurements.

Through the reported formulas, the position and orientation of a sensor relative to a fixed coordinate system (usually the Earth’s surface) were calculated. However, the “real” acceleration, even if corrected from the gravity component, could be inaccurate if the orientation of the sensor had not been well determined. To overcome this possible problem, we adopted the Kalman filter, or a complementary one. These algorithms combined the data collected through gyroscopes and accelerometers to produce a more accurate estimate of the orientation of the sensor. This corrected orientation was used to correct accelerometers data, ensuring that velocity and position integration accurately reflected the sensor movements.

#### 2.1.3. Positioning of Sensors to Collect Movements of the Hand–Wrist–Forearm System

**Figure 3:**
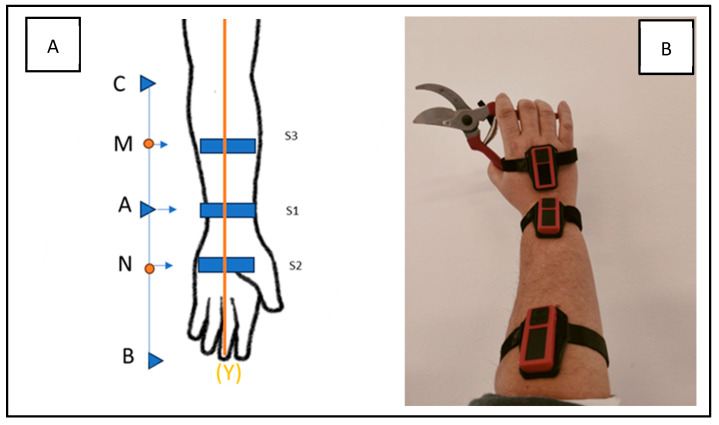
Sensor positioning methodology (**A**) and sensor placement during experimental test (**B**).

In order to collect kinetic movements of the musculoskeletal hand–wrist–forearm system, three wearable sensors were used. They were placed in three different points: forearm, wrist, and hand (as shown in Figure 3).

In order to standardize the positioning of the sensors, specific rules were followed. The first sensor (S1) was placed along the rotational axis of the wrist (A). The second one (S2) was positioned in the middle point of the AB segment (defined as the distance between S1 and the middle finger of the hand, position N). The third one (S3) was positioned in the middle point of the AC segment (defined as the distance between S1 and the elbow rotation axis, position M). All sensors were aligned along the Y-axis.

#### 2.1.4. Elaboration of Data Collected by the Three Sensors

**Figure 4:**
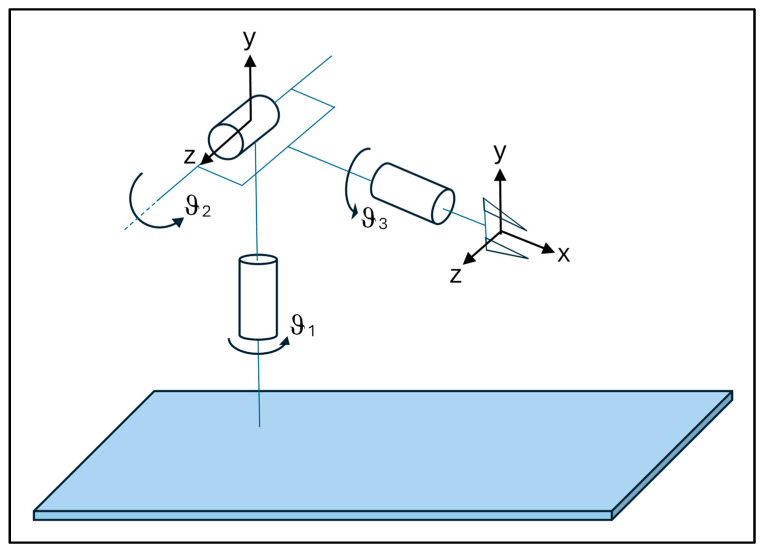
Model of the robotic arm used to study the movements of the musculoskeletal hand–wrist–forearm system. The reported angles are related to those acquired through the studied sensors.

The combination of data provided by the three sensors, applied as reported in Figure 3, allowed us to model the musculoskeletal hand–wrist–forearm system as a robotic arm which model is reported in Figure 4.

The sensors placed on the wrist and forearm were on the same axis (S2, S3—Figure 3). The sensor placed on the wrist represented the joint and it had three different axes of movement. To calculate the joint angles of a robotic arm, with a 3-axis joint, using direct kinetics, the positional and orientational relationship between the robot’s base, and its end-effector, must be evaluated on the basis of a known joint configuration. Direct kinematics, in the context of robotics, refers to the computation of the end-effector’s position and orientation (pose) from the given joint angles or positions [42].

The rotation and translation of a robotic arm involves two main types of movement: rotation (rotation around an axis) and translation (linear movement in a direction). These movements are often described using transformation matrices, which allow to express the position and orientation of each segment of the robotic arm on the basis of a reference coordinate system. This reference system is composed by a fixed coordinate system (O, x, y, z) and a mobile coordinate system (O, x’, y’, z’) that respectively represent the global reference system and the robotic arm’s reference system. Therefore, movements of the robotic arm can be decomposed into translations along the x, y, z axes and rotations around the same axes, denoted by $\theta_{x}\theta_{y}\theta_{z}$.

The translation along one of the axes can be described by the following matrix:

(5) $$T=\left[\begin{array}{cccc} 1 & 0 & 0 & dx\\ 0 & 1 & 0 & dy\\ 0 & 0 & 0 & dz\\ 0 & 0 & 1 & 1 \end{array}\right]$$

where *dx*, *dy*, and *dz* are the translation distances along the *x*, *y*, *z* axes, respectively.

Rotations around the x, y, and z axes can be described by the matrices below reported.

Rotation around the x axis:

(6) $$R_{x}\left(\theta_{x}\right)=\left[\begin{array}{cccc} 1 & 0 & 0 & 0\\ 0 & cos\left(\theta_{x}\right) & -sen\left(\theta_{x}\right) & 0\\ 0 & sen\left(\theta_{x}\right) & cos\left(\theta_{x}\right) & 0\\ 0 & 0 & 0 & 1 \end{array}\right]$$

Rotation around the y axis:

(7) $$R_{y}\left(\theta_{y}\right)=\left[\begin{array}{cccc} cos\left(\theta_{y}\right) & 0 & sen\left(\theta_{y}\right) & 0\\ 0 & 1 & 0 & 0\\ -sen\left(\theta_{y}\right) & 0 & cos\left(\theta_{y}\right) & 0\\ 0 & 0 & 0 & 1 \end{array}\right]$$

Rotation around the z axis:

(8) $$R_{z}\left(\theta_{z}\right)=\left[\begin{array}{cccc} cos\left(\theta_{z}\right) & -sen\left(\theta_{z}\right) & 0 & 0\\ sen\left(\theta_{z}\right) & cos\left(\theta_{z}\right) & 0 & 0\\ 0 & 0 & 1 & 0\\ 0 & 0 & 0 & 1 \end{array}\right]$$

The overall transformation, combining rotation and translation, can be expressed as the product of rotation and translation matrices:

(9) $$T_{rototranslation}=T\times R_{x}\left(\theta_{x}\right)\times R_{y}\left(\theta_{y}\right)\times R_{z}\left(\theta_{z}\right)$$

The above reported formulas were applied to data recorded by the three sensors in order to study the movements of the musculoskeletal hand–wrist–forearm system. Each sensor sent a string that represented data from the accelerometers and gyroscopes via Bluetooth (BLE 4.2) to an Arduino board. The data received by the Arduino board were evaluated by a software application written in Python. The sequence of steps performed by the software application were as follows:

1. Data synchronization. Sensors were read using the round robin method and each collected data was bound to a timestamp generated by the receiving system. As a consequence, all data received by the entire system were synchronized.
2. Movement calculation. According to the formulas [1,2,3,4], all movements of each sensor were calculated.
3. Angle calculation. According to the formulas [5,6,7,8,9], all movements of the robotic arm, and therefore angles of the musculoskeletal hand–wrist–forearm system, were calculated.
4. Data visualization. Angles calculated were displayed on the x, y, z axes.

The system was able to continuously collect real-time data. During the performed tests, the sampling rate was set at 50 Hz.

#### 2.1.5. Sensor Tests

All sensor tests were carried out in a laboratory. They involved four right-handed operators (O). During these tests, no object was held in the operator’s hand.

**Figure 5:**
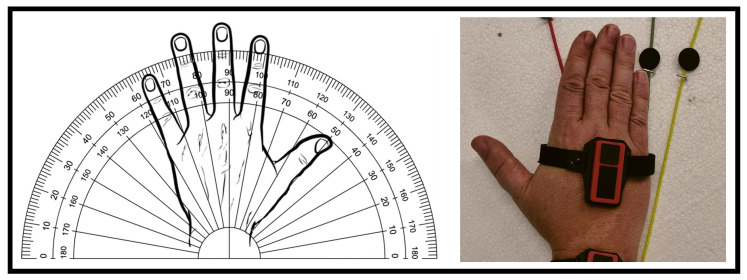
Incongruous reference positions of the musculoskeletal considered during the laboratory tests performed to evaluate the reliability of sensors.

In order to test the reliability of sensors, we used a set of incongruous positions of the musculoskeletal hand–wrist–forearm system. This set of positions was selected considering regulations, scientific literature, and ergonomic risk assessment methods. The set of positions included the 15°, 45°, and 60° angles for each axis evaluated (X, Y and Z). These positions were graphically reported on a white table by a researcher and verified by a goniometer. Each operator made the necessary movements in order to reach the selected incongruous positions (three times for each position, see Figure 5). At the end of the tests, the data recorded were compared with the reference angles adopted (i.e., 15°, 45°, and 60°, for each axis studied) [37].

A statistical procedure was used to evaluate data acquired. It was a linear mixed-effects model (procedure *lme* of the package *nlme* “Linear and Nonlinear Mixed Effects Models”—version 3.1-148) [43]. The fitted linear model was the following one:

$$Y_{ijkl}=\mu +{AN}_{i}+{AX}_{j}+AN\times {AX}_{ij}+r_{k\left(l\right)}+o_{l}+e_{ijkl}$$

where *Y* was the angle measured between the hand and the forearm, of the musculoskeletal hand–wrist–forearm system; μ was the mean; *AN_i_* was the effect of the reference angle considered (*i* = 1–3; 1 = 15°, 2 = 45°, 3 = 60°); *AX_j_* was the effect of the axis considered (*j* = 1–3; 1 = X-axis; 2 = Y-axis; 3 = Z-axis); *AN × AX_ij_* was the interaction between angle and axis considered; *r_k(l)_* was the random effect of the repetitive measurement (*k* = 1–3; nested in the operator (*l* = 1–4); *o_l_* was the random effect of the operator (*l* = 1–4) and *e_ijkl_* was the residual error. Furthermore, an autoregressive covariance structure (correlation = corAR1 [44]) was used to account for the repeated measurements on the same operator.

### 2.2. Phase 2: Application of the Developed Measurement System to a Real Case

#### 2.2.1. Field Test Design

After having developed and tested all the sensors, the resulting data acquisition system was applied to a real case (i.e., the pruning of a vine). All four operators involved in the tests were right-handed (although the system can work also with left-handed operators).

**Figure 6:**
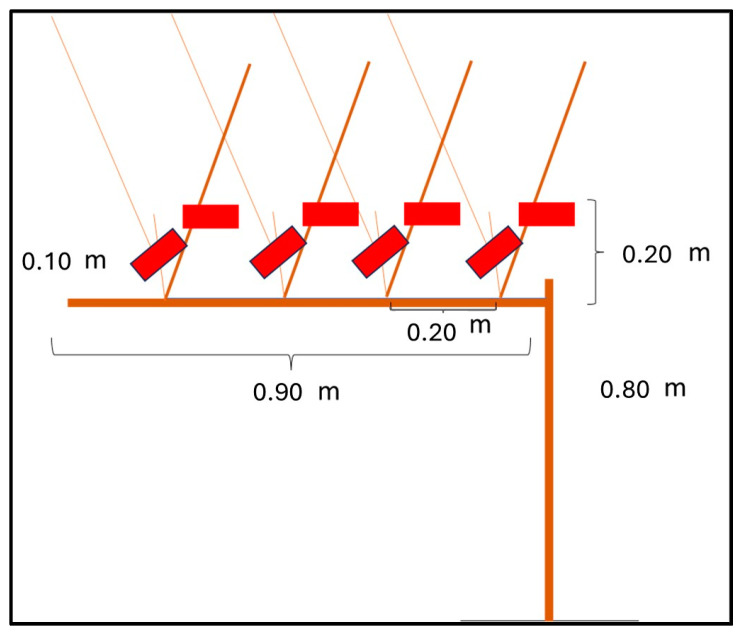
Simulation of the vineyard and cuts. The brown structure is the simulated grapevine used for the performed cutting tests; the red parts show the cutting sequence, performed three times by each operator involved in the tests.

A vine, with a form of spurred cordon pruning, was simulated in a laboratory [27,28] in order to standardize the cutting procedure (Figure 6). The “experimental vine” reproduced the dimensions of a real vine and gave us the opportunity to simulate realistic pruning cuts. In the “experimental vine”, different vine branches were simulated with electric cables while a plastic support was used as the trunk of the vine. Positions of the vine branches (i.e., the pruning cuts to do) were derived from literature that regards the spurred cordon pruning [13].

The cutting procedure consisted of seven cuts to do in sequence. When a cutting procedure was concluded by an operator, a researcher rebuilt the vine branches that had been cut in order to allow the starting of a new cutting procedure. Each operator performed a complete cutting procedure three times. All performed cutting procedures were recorded by the data acquisition system.

#### 2.2.2. Evaluation of Data Recorded in Field Tests

**Figure 7:**
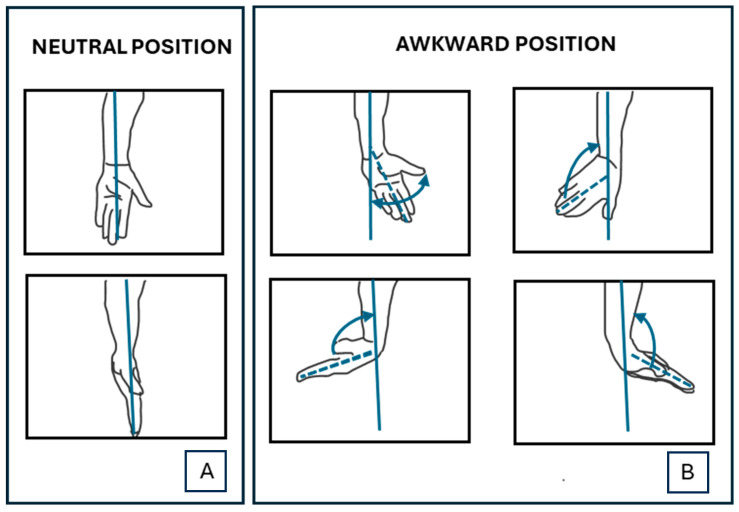
Graphical examples of usual positions (**A**) and limit angles (**B**) identified as risk factors on the basis of regulations, scientific literature, and ergonomic risk assessment methods. If these awkward postures (**B**) are assumed many times during a workday, for a long period, they could cause an occupational disease.

Data recorded in field tests were evaluated to quantify possible risk factors for the operators. Risk factors were calculated considering different risk thresholds (i.e., different limit angles for the hand–wrist–forearm system—Figure 7) identified on the basis of regulations, scientific literature, and ergonomic risk assessment methods.

On the basis of the identified risk thresholds, risk factors were classified as follows, in accordance to levels of the international analysis methods [18]:

- **low level:** the operator does not assume awkward postures for a long time, and no anomalous behaviors that could cause occupational diseases are found. This level is represented by the color green and corresponds to a low and acceptable risk.
- **medium level**: it is necessary to carefully monitor the worker. Some inappropriate postures are detected, which could generate occupational diseases in the future. This level is represented by the yellow threshold and represents moderate risk.
- **high level**: there is an incorrect working condition. The worker assumes incongruous positions for prolonged time. The risk level is represented by the color red and corresponds to a high and unacceptable risk level, which produces occupational diseases.

## 3. Results

### 3.1. Phase 1: Development and Testing of the Measuring System

In the first phase of the research, the sensors were developed and tested in a laboratory. Four operators (O) were involved in the tests. Each operator moved his arm to a specific set of reference positions three times (Figure 5). As far as angles and axes are concerned, the data recorded by the sensors being tested are reported in Table 1.

On a data recorder during laboratory tests, a statistical analysis was performed in order to evaluate the reliability of sensors. For each reference position considered, the mean value of angles, recorded by the sensors, was calculated and statistical significance, between mean values obtained was determined. In Table 2, mean values and statistical significance of the mean are reported.

### 3.2. Phase 2: Application of the Measurement System Developed on a Real Case

In a following phase of the experiment, we recorded and analyzed the movements necessary for the pruning of a vine. Four operators were involved in the tests. Each operator made a sequence of seven cuts to an experimental vine, repeated three times. For each sequence of cuts, accelerations of the musculoskeletal hand–wrist–forearm system, recorded by the sensors, were graphically evaluated to detect the time span connected to the cuts performed. On the basis of temporal windows identified, and data flows recorded by the sensors, the angles shown by the musculoskeletal hand–wrist–forearm system were estimated for the whole sequence of cuts, as reported in Table 3 for all the operators involved in the test.

In a final phase of the research, the estimated angles for the movements by the operators were compared with specific risk thresholds and classified in terms of low-, medium-, and high-risk factors. The incidence of each level, on all the simulations performed, is shown in Table 4.

## 4. Discussion

### 4.1. The Developed Sensor

The first key requirement for the sensor development was to reach a good relationship between cost and performance. The hardware selected for the development of the sensor cost around 35 € and it allowed us to build a whole data acquisition system with a limited investment. During our laboratory tests, the reliability of the sensor was evaluated through a set of reference positions and for the X, Y, and Z cardinal axes. The results obtained showed a good relationship between the reference positions evaluated and the angles measured by the sensor (*p* < 0.01), without any statistical difference between the cardinal axes investigated (*p* = 0.88). As a consequence, the first fundamental requirement for the sensor development was considered to be fulfilled.

Other requirements considered during the system development were to obtain good robustness and “wearability” of the sensor in order to record data of movement in different parts of the body, particularly the upper limbs, in a reliable and easy way. To reach these goals, a dedicated case was studied and a band was added to the sensor. The good results obtained both in the laboratory and during the simulated field tests by placing three sensors on different parts of an upper limb (hand, wrist, and forearm) and when some occasional collisions happened, proved that these requirements were also fulfilled.

### 4.2. The Developed Measuring System Applied to Conventional Risk Assessment Methods

Currently, the available methods for the assessment of risks pertaining to uncomfortable working postures are based on indirect analyses. These indirect analyses consist of the evaluation of pictures, or moves, and can experience some errors or unreliable data.

The data acquisition system that has been developed showed a good reliability in the measurements of movements performed by the musculoskeletal hand–wrist–forearm system. Even if a direct comparison with conventional methods for risk assessment [45], such as RULA, OCRA, or REBA, was not performed in this study, we think that the data collected through the measuring system developed could allow us to calculate the following indexes in a more efficient way:

RULA

- wrist inclination;
- inclination angle;
- level of risk during the work phases.

OCRA

- frequency of the repeated gesture;
- number of gestures per work cycle;
- worker break times;
- sequential pattern.

REBA

- arm position;
- wrist position.

As a consequence, the availability of these indexes in real time could allow an automatic risk assessment, permitting us to give back to the operator fast ergonomic feedback both in work and training activities [46,47].

Furthermore, the developed data acquisition system was able to record upper limb movements in the space without the need for absolute coordinates (such as those that could be given by a GPS system). This opportunity, and the ability to collect data for some hours, could let it be employed in all open fields of operation and allow the calculation of risk assessment indexes in a more effective way. Further investigations will check the feasibility of this hypothesis.

### 4.3. The Developed Measuring System Applied to the Pruning of a Vine

The previously cited systems, such as the ZurichMOVE 1, Delsys Trigno, and Novel Pedar, have been broadly applied in many medical and industrial sectors. However, they are not specifically focused on agricultural tasks, and seldom have they been tested in similar field conditions. In fact, as reported by Lind et al. [48] in a systematic review on wearable motion capture devices carried out in 2023, conventional IMUs, similar to those developed in this research, were shown to be expensive and not mechanically adequate to work activity measurement [48]. Furthermore, no studies were found where IMUs have been adopted to acquire wrist angles in a real scenario [48].

Instead, the monitoring system developed in this study proved to be well tailored for agricultural activities, in particular for viticultural tasks. For the development of the sensors, some low-cost and open-source hardware components were chosen. This allowed us to build a cheap measurement system, usable on a large number of vine operators and potentially replicable by other researchers for human movement studies [48].

In the laboratory tests, the measuring system showed a good relationship between the evaluated reference positions and the wrist angles acquired by the sensors for each cardinal axis investigated. Similar results have been achieved by other authors too, when wearable sensors based on similar IMUs have been evaluated. For example, evaluating new IMUs to monitor wrist flexion velocity, Manivasagam and Liyun [49] found a small bias the moment they compared the recorded data to those acquired through a goniometer. Moreover, considering a network of wearable IMUs to acquire a worker’s upper body movements, Vignais et al. [46] found that the joint angles of the biomechanical model connected to the network of sensors were automatically estimated in real time.

Not only did the developed sensors show a reliable measurement of kinetic data, but also a good robustness and wearability. Generally, similar systems require the assistance of a second person for the installation, setting, and monitoring [50]. Thus, they are not immediately wearable and usable in a real workplace [50]. After this preliminary study, with some further developments, and for some coded activities, the system developed could be worn and used independently by any operator. As a result, an efficient monitoring of farming activities could be allowed, often non-standardized and significantly influenced by external factors such as plant layout, weather conditions, humidity, and mud. Instead, other systems based on IMUs or surface electromyography (EMG), the two main technologies adopted to measure human’ movements across various joints [51,52], are primarily focused on laboratory conditions or indoor applications. Therefore, the system developed could facilitate the understanding of the biomechanical risks associated with repetitive and strenuous agricultural tasks. As a consequence, it could lead to the development of more effective strategies for injury prevention, improving workers’ safety and productivity in agriculture.

When applied to simulated vineyard tasks, the system collected useful data, demonstrating the presence of risks in the pruning of a vine. Incongruous gestures, considering all types of movements (i.e., all X, Y, and Z cardinal axes), corresponded to ~40% of the total movements performed by the operators involved in the tests. This result can be considered in line with similar studies [53,54,55,56] carried out on specific biomechanical risks in agriculture [57,58]. For example, in a study conducted by INAIL (National Institute for Insurance against Workplace Accidents) [57] the technical gesture of pruning was assessed through the OCRA index and evaluated as an extremely dangerous movement, both in frequency and intensity, with a risk scored above 22.6 (maximum risk factor). A similar result was achieved in a study by Montomoli et al. [59], where the activity of pruning was classified as an even higher value of risk (i.e., of 27), once again through the OCRA methodology.

Incongruous repetitive movements represent a clear risk for operators’ health. Studies on carpal tunnel syndrome demonstrated that in 61% of cases, such pathology is caused by the improper posture of the joints and the frequency of the gesture [60,61]. Also You, in 2013 [62], analyzing previous studies across different productive sectors, showed that the limit of 45° in the wrist joint is frequently exceeded in various types of repetitive activities, particularly in the agricultural sector [63,64,65,66,67], as it was in this preliminary study.

However, in the “experimental vine” used in the tests, no leaves or wires were simulated. Leaves and wires could increase the rotation of the hand–wrist–forearm up to 30% of the time and expose workers to further dangerous postures. As a consequence, it is necessary to assume that maximum risk levels in a real case could be higher than those estimated in this preliminary investigation.

### 4.4. Future Improvements

The calculation of the angles described by the musculoskeletal system under study required a graphical evaluation of the recorded accelerations in order to identify the moment of the cutting action performed. In the study of the pruning of a vine, this fact did not affect the effectiveness of data collected. Nevertheless, with more complex and continuous movements, this could partially limit the applicability of the data recording system. Some further research will be needed to test the sensors with more complex movements and improve the software algorithm in order to automatically detect the cutting action performed.

In this preliminary study, the indexes to evaluate risk assessment by conventional methods, such as RULA, OCRA, and REBA, have not been considered. In a possible future improvement of the system, all processing of collected raw data could be embedded in the hardware used to build the system. As a consequence, the system could produce real-time risk assessment estimations for some coded movements. Furthermore, through signals of different colors displayed by the sensor monitors, the system could give ergonomic feedback to operators in order to correct possible incongruous postures or movements, both on the worker and in the training applications.

The current sensors’ sampling rate allows a continuous monitoring of work activities for up to 6 h. Therefore, the developed system in its current setup and hardware layout does not allow us to monitor a full 8-h workday. Further improvements to the system setup and hardware layout will be useful to overcome this limitation allowing a continuous monitoring in a real-time scenario, as already happens with many smart wearable sensors used for the tracking of human health [51,52,68].

## 5. Conclusions

Ergonomics in agriculture is an interdisciplinary topic that involves the identification of risk factors related to MSDs, the determination of causes, as well as the development and implementation of possible ergonomic interventions. The developed monitoring system has shown good reliability in the measurements of the main parameters related to the movements of a musculoskeletal hand–wrist–forearm system. When applied to study a possible real case, such as the pruning of a vine, it allowed us to evaluate a simulated sequence of pruning and to quantify the levels of risk induced by this type of viticultural activity. Therefore, the developed system has proven to be capable of increasing the operator’s safety level in the vineyard, and it can also be used to study other typical operations of viticulture, such as the harvesting or unloading of grapes in wine cellars. Furthermore, other agricultural sectors could also find this new system interesting, for example, the oil production chain.

## Figures and Tables

**Table 1:** Angles measured by the sensors in different reference positions (defined by angles and axes) and with different operators.

Angle	Axis	Operator											
		O_1_			O_2_			O_3_			O_4_		
		R1	R2	R3	R1	R2	R3	R1	R2	R3	R1	R2	R3
15°	X	15.30°	14.99°	15.23°	15.18°	15.22°	15.00°	14.97°	15.20°	15.01°	15.00°	15.02°	15.01°
	Y	14.98°	15.07°	14.95°	15.30°	15.20°	15.15°	15.02°	15.15°	14.97°	15.30°	15.01°	14.98°
	Z	15.06°	15.15°	14.97°	15.21°	15.11°	14.98°	15.00°	15.00°	15.00°	14.96°	15.01°	15.02°
45°	X	45.01°	44.90°	45.03°	45.01°	45.10°	44.98°	45.06°	45.03°	45.00°	45.10°	44.97°	45.01°
	Y	44.99°	45.10°	45.10°	45.00°	49.80°	45.02°	45.00°	45.01°	45.10°	45.01°	45.50°	45.00°
	Z	44.98°	45.07°	45.03°	44.95°	45.00°	44.90°	45.10°	45.20°	45.10°	45.00°	44.99°	45.10°
60°	X	59.78°	60.10°	60.50°	59.98°	60.00°	60.01°	60.10°	60.11°	60.11°	59.88°	60.40°	60.01°
	Y	60.10°	59.97°	60.12°	60.20°	60.09°	59.98°	59.99°	60.01°	60.11°	60.09°	60.20°	60.02°
	Z	59.97°	60.07°	60.34°	60.15°	60.00°	60.10°	60.10°	59.96°	60.01°	60.01°	60.02°	60.15°

**Table 2:** Mean values and standard deviations of angles measured by the sensors in different reference positions (defined by angles and axes).

Angles	Axes			
	X (Avg. ± S.D.)	Y (Avg. ± S.D.)	Z (Avg. ± S.D.)	All (Avg. ± S.D.)
15°	15.09° ± 0.12 ^A^	15.09° ± 0.13 ^A^	15.04° ± 0.08 ^A^	15.07° ± 0.11 ^A^
45°	45.02° ± 0.06 ^B^	45.47° ± 1.37 ^B^	45.04° ± 0.08 ^B^	45.17° ± 0.80 ^B^
60°	60.08° ± 0.20 ^C^	60.07° ± 0.08 ^C^	60.07° ± 0.11 ^C^	60.07° ± 0.13 ^C^

A,B,C means in the same column with different uppercase superscripts significantly differ (*p* < 0.01).

**Table 3:** Angles estimated for the musculoskeletal hand–wrist–forearm system of four operators. during three sequences of seven cuts each, performed on the experimental vine.

Cutting	Axis	Operator											
		O1			O2			O3			O4		
		Cutting Sequence			Cutting Sequence			Cutting Sequence			Cutting Sequence		
		S1	S2	S3	S1	S2	S3	S1	S2	S3	S1	S2	S3
1	X	00.56°	15.21°	22.23°	18.47°	39.45°	39.28°	48.08°	46.11°	02.13°	00.18°	00.23°	00.15°
	Y	46.40°	50.16°	31.24°	49.00°	60.11°	55.27°	49.88°	47.90°	19.20°	33.16°	02.50°	45.70°
	Z	45.11°	10.61°	27.85°	30.16°	15.84°	22.26°	03.71°	10.28°	14.65°	47.80°	46.00°	47.84°
2	X	00.45°	10.37°	00.47°	11.21°	00.55°	33.24°	23.26°	23.64°	48.16°	23.45°	22.43°	49.58°
	Y	00.00°	03.00°	14.00°	47.11°	46.00°	47.34°	46.23°	47.11°	50.11°	33.07°	33.08°	44.01°
	Z	10.11°	23.15°	15.30°	20.10°	22.17°	10.21°	10.33°	08.45°	20.70°	25.50°	30.80°	48.90°
3	X	00.10°	15.89°	49.00°	45.71°	48.34°	44.16°	35.28°	45.36°	03.70°	04.87°	17.50°	23.34°
	Y	15.33°	23.44°	33.18°	47.18°	49.55°	33.25°	36.88°	47.10°	50.02°	33.00°	48.80°	45.80°
	Z	47.00°	10.70°	11.23°	48.55°	49.74°	50.22°	55.00°	60.00°	15.20°	49.70°	34.17°	46.11°
4	X	02.11°	03.10°	00.66°	22.12°	33.10°	13.44°	10.24°	23.22°	32.44°	47.70°	12.34°	33.60°
	Y	33.00°	45.60°	59.10°	47.00°	48.50°	45.50°	50.00°	48.30°	47.40°	09.00°	47.40°	45.00°
	Z	47.33°	39.00°	11.14°	15.56°	47.88°	50.10°	49.33°	45.60°	47.60°	48.50°	48.45°	49.00°
5	X	29.22°	13.00°	09.05°	00.15°	12.44°	22.16°	00.22°	11.33°	25.50°	12.56°	09.21°	20.05°
	Y	37.15°	44.34°	27.30°	47.40°	35.21°	35.30°	55.00°	48.30°	55.00°	47.33°	38.11°	30.44°
	Z	15.29°	13.34°	04.22°	09.56°	45.70°	36.00°	50.10°	46.80°	47.00°	45.80°	46.32°	38.00°
6	X	01.12°	00.25°	22.30°	12.88°	02.99°	34.78°	24.50°	20.68°	23.44°	13.21°	02.33°	23.44°
	Y	47.40°	46.40°	45.70°	49.50°	47.80°	23.45°	31.00°	25.60°	43.00°	33.30°	51.00°	49.60°
	Z	47.33°	46.13°	46.00°	09.34°	48.01°	12.00°	54.20°	45.80°	47.40°	49.40°	46.88°	48.00°
7	X	10.13°	00.85°	02.76°	11.50°	00.00°	11.56°	02.61°	10.33°	10.45°	03.43°	04.15°	33.18°
	Y	45.88°	55.00°	48.70°	53.20°	30.00°	47.40°	48.00°	39.13°	55.22°	05.23°	20.13°	22.03°
	Z	45.80°	05.11°	47.40°	55.80°	10.20°	10.66°	10.15°	15.28°	49.00°	47.88°	45.15°	23.08°

**Table 4:** Frequency of angles oh the musculoskeletal hand–wrist–forearm system, estimated for four operators, during three sequences of seven cuts each, performed on the experimental vine. The angles shown here below have been classified in terms of low, medium, and high risk on the basis of the international analysis methods.

Levels of Risk		Angles	Axes			
			X	Y	Z	All
High level		>45	10%	57%	52%	40%
Medium level: uncertain. Potentially dangerous risk level		20–45	33%	35%	18%	28%
Low level: appropriate position		0–20	57%	8%	30%	32%

## Data Availability

Data are contained within the article.

## Abbreviations

MSD	Musculoskeletal Disorders
MAPO	Method to Assess the Risk of Patient Manual Handling in Hospital Wards
NIOSH	National Institute for Occupational Safety and Health method
OCRA	Occupational Repetitive Action
REBA	Rapid Entire Body Assessment
RULA	Rapid Upper Limb Assessment
